# Immunoinformatic prediction to identify *Staphylococcus aureus* peptides that bind to CD8+ T-cells as potential vaccine candidates

**DOI:** 10.14202/vetworld.2024.1413-1422

**Published:** 2024-06-28

**Authors:** Grisilda Vidya Bernhardt, Kavitha Bernhardt, Pooja Shivappa, Janita Rita Trinita Pinto

**Affiliations:** 1 Department of Biochemistry, RAK College of Medical Sciences, RAK Medical and Health Sciences University, Ras Al Khaimah, United Arab Emirates; 2 Department of Basic Medical Sciences, Division of Physiology, Manipal Academy of Higher Education, Manipal, Karnataka, India; 3 Department of Biomedical Sciences, College of Medicine, Gulf Medical University, Ajman, United Arab Emirates

**Keywords:** autologous *Staphylococcus* lysate therapy, CD8+ T-cell immunity, computational tools, epitopes, immunoinformatics, immunological responses, major histocompatibility complex class I binding epitopes, molecular docking simulations, *Staphylococcus aureus*, vaccine development

## Abstract

**Background and Aim:**

*Staphylococcus aureus*, with its diverse virulence factors and immune response evasion mechanisms, presents a formidable challenge as an opportunistic pathogen. Developing an effective vaccine against *S. aureus* has proven elusive despite extensive efforts. Autologous *Staphylococcus* lysate (ASL) treatment has proven effective in triggering an immune response against bovine mastitis. Peptides that stimulate the immune response can be the subject of further research. The study aimed to use immunoinformatics tools to identify epitopes on *S. aureus* surface and secretory proteins that can bind to major histocompatibility complex class I (MHC I) and CD8+ T-cells. This method aids in discovering prospective vaccine candidates and elucidating the rationale behind ASL therapy’s efficacy.

**Materials and Methods:**

Proteins were identified using both literature search and the National Center for Biotechnology Information search engine Entrez. Self and non-self peptides, allergenicity predictions, epitope locations, and physicochemical characteristics were determined using sequence alignment, AllerTOP, SVMTriP, and Protein-Sol tools. Hex was employed for simulating the docking interactions between *S. aureus* proteins and the MHC I + CD8+ T-cells complex. The binding sites of *S. aureus* proteins were assessed using Computer Atlas of Surface Topography of Proteins (CASTp) while docked with MHC I and CD8+ T-cells.

**Results:**

Nine potential *S. aureus* peptides and their corresponding epitopes were identified in this study, stimulating cytotoxic T-cell mediated immunity. The peptides were analyzed for similarity with self-antigens and allergenicity. 1d20, 2noj, 1n67, 1nu7, 1amx, and 2b71, non-self and stable, are potential elicitors of the cytotoxic T-cell response. The energy values from docking simulations of peptide-MHC I complexes with the CD8+ and T-cell receptor (TCR) indicate the stability and strength of the formed complexes. These peptides – 2noj, 1d20, 1n67, 2b71, 1nu7, 1yn3, 1amx, 2gi9, and 1edk – demonstrated robust MHC I binding, as evidenced by their low binding energies. Peptide 2gi9 exhibited the lowest energy value, followed by 2noj, 1nu7, 1n67, and 1d20, when docked with MHC I and CD8 + TCR, suggesting a highly stable complex. CASTp analysis indicated substantial binding pockets in the docked complexes, with peptide 1d20 showing the highest values for area and volume, suggesting its potential as an effective elicitor of immunological responses. These peptides – 2noj, 2gi9, 1d20, and 1n67 – stand out for vaccine development and T-cell activation against *S. aureus*.

**Conclusion:**

This study sheds light on the design and development of *S. aureus* vaccines, highlighting the significance of employing computational methods in conjunction with experimental verification. The significance of T-cell responses in combating *S. aureus* infections is emphasized by this study. More experiments are needed to confirm the effectiveness of these vaccine candidates and discover their possible medical uses.

## Introduction

*Staphylococcus aureus* functions as a versatile and opportunistic Gram-positive cocci. This bacterium can infect, invade, persist, and replicate extensively within various human tissues, including the skin, bones, organs, and vasculature. About 30% of people generally harbor *S. aureus* in their anterior nares, a major source for the organism’s spread to other parts of the body [1, 2]. With various virulence factors, including invasins, protein A, capsule coating, clotting factors, hemolysins, leukotoxin, and leukocidins, *S. aureus* is capable of causing a wide spectrum of infections ranging from localized skin infections to life-threatening bacteremia and septic shock [1, 3]. Phagocytes usually eliminate extracellular *S. aureus* by engulfing it. CD4+ T lymphocytes aid bacterial elimination by activating B lymphocytes to produce antibodies and recruiting phagocytes, such as macrophages and neutrophils, to the infection site. *S. aureus* can also persist intracellularly when it escapes from the phagosome into the cytoplasm. CD8+ T-cells and natural killer cells are the specific immune cells responsible for eliminating intracellular *S. aureus* to prevent subsequent harm [4, 5]. *S. aureus* can evade immune detection by disguising itself in a capsule or confusing the immune system through antigen masking [6]. *S. aureus* increases its resistance to immune attacks by forming biofilms and directly inhibiting phagocytosis [6–8]. This evasion can make infections persistent and difficult to treat with antibiotics, particularly when *S. aureus* hides inside immune cells such as the neutrophils. Opsonin-dependent phagocytosis plays a pivotal protective role against staphylococcal infections, and deficiencies in opsonin generation by phagocytes are linked to increased susceptibility to infections [9–11].

Despite global research efforts, an effective vaccine against *S. aureus* infections remains elusive [5, 12–14]. While stimulating cell-mediated immunity and treating infections effectively, the mechanisms underlying autologous *Staphylococcus* lysate (ASL) therapy remain elusive and require further investigation [15–18]. For bovine mastitis and other staphylococcal infections, an innovative alternative therapy involves the use of ASL. The vaccine is personalized using *S. aureus* strains obtained from the patient. The patient is given a dose of the inactivated bacterial ASL vaccine to enhance their immune response. Although promising in stimulating cell-mediated immunity and effectively treating infections, the exact mechanisms by which ASL therapy works are still being researched [15–18]. The interactions between *S. aureus* proteins and immune cells, specifically how these proteins can stimulate CD4+ T-cells (helper T-cells that coordinate the immune response) and CD8+ T-cells (cytotoxic T-cells responsible for destroying infected cells), can be studied using immunoinformatics tools [14, 19]. This study hypothesizes that specific proteins of *S. aureus* may enhance the CD8+ T-cell response, aiding in the elimination of intracellular bacteria. Advances in genome sequencing technology have dramatically increased the number of bacterial genomes available for study, providing an excellent opportunity to identify potential targets for vaccines or therapeutics through computational biology methods, such as reverse vaccination and comparative genomics [5, 14]. Screening for major histocompatibility complex class I (MHC I) molecules and CD8+ T-cells through bioinformatics techniques holds advantages due to high MHC I variability [14, 15]. This approach enables the identification of bacterial proteins that could trigger a strong immune response by presenting antigens on the surface of cells for recognition by CD8+ T-cells. Surface bacterial proteins, easily accessible for host cell attachment and immunogenic, are excellent vaccine candidates [20, 21]. These potential therapeutic targets must not provoke adverse reactions by triggering responses against the host’s tissues [19, 20, 22]. Previous research [23–27] have shown that specific *S. aureus* antigens can elicit robust immune reactions in animals, evidenced by the production of protective antibodies against harmful *S. aureus* strains. These antigens have not effectively induced a protective response in human trials, highlighting the need for further investigation into CD8+ T cell-mediated immunity for successful vaccine development.

This study employed immunoinformatics tools to analyze *S. aureus* surface and secretory proteins for their ability to interact with CD8+ T-cell receptors through binding to MHC Class I molecules. These methods can uncover novel vaccine candidates by revealing the molecular interactions between bacterial antigens and the host immune system. This study also investigates the contributing mechanisms of ASL treatment’s success against staphylococcal infections.

## Materials and Methods

### Ethical approval

This study was approved by the Institutional Review Board of the RAKMHSU (approval no. RAKMHSU-REC-191-2020-FM).

### Tools used

Amino acid sequences of the surface and secretory proteins of *S. aureus* were retrieved based on a literature search from National Center for Biotechnology Information (NCBI) protein sequence database using the identification numbers (IDs) of the proteins. The NCBI is part of the United States National Library of Medicine, a branch of the National Institutes of Health (Available at https://www.ncbi.nlm.nih.gov/) [28].

To identify sequence similarities between *S. aureus* proteins and mammalian MHC I protein complexes, we employed the multiple sequence alignment tool Clustal Omega (ClustalO) (available at http://www.clustal.org/omega/). This tool determines pairwise alignments between sequences and determines degrees of similarity [29, 30].

To predict the antigenicity, allergenicity, solubility, and physicochemical characteristics of selected proteins, we used the following tools:

AllerTOP for predicting allergens (available at https://www.ddg-pharmfac.net/AllerTOP/).

Protein-Sol for predicting solubility (available at https://protein-sol.manchester.ac.uk/).

SVMTriP for predicting the linear antigen epitopes of *S. aureus* proteins relevant to MHC I [31].

Hex 5.0 was used for molecular docking simulations, employing spherical polar Fourier correlation for efficient docking and superimposition calculations between protein molecules or between a protein and a ligand (available at https://hex.loria.fr/). Hex also computes and presents viable docking models that provide insight into energy minimization values and binding affinities [32, 33].

DeepView – Swiss-PdbViewer for comparing active sites and energy minimization (available at https://spdbv.unil.ch/). This application analyzes multiple proteins simultaneously through structural alignments and a comparison of active sites [34].

The computational server, Computer Atlas of Surface Topography of Proteins (CASTp), was used to identify suitable binding pockets in docked structures and verify their antigenicity (available at http://sts.bioe.uic.edu/castp/index.html?2pk9).

CASTp measures and finds surface-accessible pockets and interior-inaccessible cavities for proteins and other molecules using the pocket algorithm to analyze appropriate binding regions. It uses weighted Delaunay triangulation and the alpha complex for shape measurement [35].

### Methodology

#### Identification of relevant proteins

We identified the probable antigenic surface proteins of *S. aureus* using the NCBI search engine Entrez. The sequences in FASTA format of all selected antigenic proteins of *S. aureus* and the MHC I molecule binding domain, along with CD8+ and T-cell receptor (TCR), were obtained from NCBI using their respective accession IDs [28].

#### Identification of human self-peptides, prediction of epitopes, and physicochemical characteristics

We used ClustalO to identify regions of high conservation between the selected surface protein and MHC I. For further analysis, antigenic protein sequences of *S. aureus* with the lowest degree of similarity, indicating the lowest energy scores, were chosen for docking simulation with MHC I + CD8+ and TCR using Hex. Energy scores reveal regions of high conservation between the *S. aureus* proteins and MHC I [29]. These sequences were submitted in the FASTA format for epitope prediction, generating every conceivable 20-mer peptide from each acquired *S. aureus* protein. Each peptide’s expected binding affinities to MHC I was predicted [30, 31].

#### Molecular docking simulation, energy minimization, and assessment of binding pockets

For the docking simulations, we loaded the FASTA sequence of the selected *S. aureus* antigenic peptides and the mammalian MHC I receptor protein (GenBank ID AFR73979.1, PDB23144384) into Hex. The scoring algorithms used in Hex allowed us to predict the strongest binding interactions between the selected peptides and MHC I based on score analysis. The following parameters were used to perform the docking analysis. Search mode: Full rotation; Correlation type: Shape only; Receptor range: 180; Ligand range: 180; Samples: 642; Twist range: 30; Sampling rate: 128/min; Distance range: 30; Grid dimension: 0.6; Steric scan: 16; Final search: 1. To predict the interactions between stable docked structures of MHC I and *S. aureus* proteins with CD8+ and TCR, the selected antigenic peptides from *S. aureus* and MHC class I were loaded into Hex as ligands, whereas CD8+ and TCR were designated as receptors. During the docking process, water molecules located more than 5 Å away from the active site residues were removed from the crystal structures, and polar hydrogen molecules were added. Subsequently, the protein structure was minimized using a force field until the minimized structure’s root mean square deviation was ≤0.30 Å compared to the crystal structure [36].

Subsequent steps involved energy minimization and evaluation of the binding pockets’ area and volume in the docked structures using CASTp [35].

## Results

### Identification of potential S. aureus peptide epitopes for T-cell immunity through MHC I

We identified and predicted the epitopes of selected *S. aureus* peptides for MHC I using the SVMTriP tool. The predictions are summarized in Table-1; all selected epitopes had a recommended score of 1 and a set epitope length of 20 amino acids. The protein data bank (PDB) IDs of the proteins, alignment scores from the multiple sequence alignments of MHC Class I with selected proteins along with their antigenicity, stability index, amino acid count, predicted solubility, and additional characteristics derived from our analysis, are presented in Table-2.

**Table-1:** MHC 1- CD8+T-cell epitope antigenic candidates based on SVMTriP MHC 1 prediction.

ID	Protein sequence used for predication	Location	Epitope	Score
1amx	MRGSHHHHHHGSITSGNKSTNVTVHKSEAGTSSVFYYK	92–111	HSNYYSGQSAITDFEKAFPG	1.000
	TGDMLPEDTTHVRWFLNINNEKSYVSKDITIKDQIQGGOO			
	LDLSTLNINVTGTHSNYYSGOSAITDFEKAFPGSKITVDNT			
	ENTIDVTIPQGYGSYNSFSINYKTKITNEQQKEFVNNSQAW			
	YQEHGKEEVNGKSFNHTVHN			
1yn3	GSTVPYTITVNGTSQNILSNLTENKNONISYKDLEGKVKSVL	66–85	KNGTKKVIDLKSGIYTANLI	1.000
	ESNRGITDVDLRLSKQAKYTVNFKNGTKKVIDLKSGIYTA			
	NLINSSDIKSININID			
1nu7	IVEGSDAEIGMSPWQVMLFRKSPOELLCGASLISDRW	215–234	FNNRWYQMGIVSWGEGCDRD	1.000
	VLTAAHCLLYPPWDKNETENDLLVRIGKHSRTRYERNIEK			
	ISMLEKIYIHPRYNWRENLDRDIALMKLKKPVAFSDYIHPVCL			
	PDRETAASLLQAGYKGRVTGWGNLKETWTANVGK			
	GQPSVLOVVNLPIVERPVCKDSTRIRITDNMFCAGYKPDEGKRGDA			
	CEGDSGGPFVMKSPFNNRWYOMGIVSWGEGCDR			
	DGKYGFYTHVFRLKKWIQKVIDQFGE			
2b71	MGWSCIILFLVATATGVHSQVQLQHSGGGLEQPGGSLR	180–199	ALTSGVHTFPAVLOSSGLYS	1.000
	ISCAASGETENTNDMSWVRQAPGKGLOWVSTIIGIDD			
	TTHYADSVRGRETVSRDTSKNMVYLQMNSLRVEDTALY			
	YCVKNSGIYSFWGQGTLVTVSSASTKGPSVFPLAPSSK			
	STSGGTAALGCLVKDYFPEPVTVSWNSGALTSGVHTFPA			
	VLOSSGLYSLSSVVTVPSSSLGTQTYICNVNHKPSNKV			
	DKKVEPKSCDKTHT			
1edk	AQHDEAQQNAFYQVLNMPNLNADORNGFIQ	5–24	EAQQNAFYQVLNMPNLNADQ	1.000
	SLKDDPSQSANVLGEAQKLNDSQAPK			
2gi9	MOYKLILNGKTLKGETTTEA	23–42	AATAEKVFKQYANDNGVDGE	1.00
	VDAATAEKVFKQYANDNGVDGEWTYDDATKTFTVTE			
1n67	MRGSHHHHHHGSLVPRGSMVAADAPAAGTDIT	139–158	DPENVKKTGNVTLATGIGST	1.00
	NQLTNVTVGIDSGTTVYPHQAGYVKLNYGFSVPN			
	SAVKGDTFKITVPKELNLNGVTSTAKVPPIMAGD			
	QVLANGVIDSDGNVIYTFTDYVNTKDDVKATLTM			
	PAYIDPENVKKTGNVTLATGIGSTTANKTVLVDYEK			
	YGKFYNLSIKGTIDQIDKTNNTYROTIYVNPSGDNVIA			
	PVLTGNLKPNTDSNALIDOONTSIKVYKVDNAADLSESY			
	FVNPENFEDVINSVNITFPNPNOYKVEFNTPDDQITTPYIV			
	VVNGHIDPNSKGDLALRSTLYGYNSNIIWRSMSWDNEV			
	AFNNGSGSGDGIDKPVVPEQPDEPGEIEPIPEK			
2noj	QTKNVEAAKKYDQYQTNEKKQVNKKVVDAQKAVELF	23–42	NKKVVDAQKAVELFKRTRTV	1.00
	KRTRTVATHRKAQRAVNLIHFQHSYEKKKLQRQIDLVLKYNTLK			
1d20	MKNKVLKFMVFIMLLNITPLFNKNDAFAARDISS	555–576	EKVSVNLLANGEKVKTVDVT	1.00
	NTNVDLTVPSQPKIEDGGKTVTVMTFDFDENGI			
	KIQNGDTIKVAFPITSGWTIEGSKYTVTLPVKGEQVG			
	AQVATIPDGATITFNNDKVELISDVGSPFAEFEVQG			
	RNLIQTNTSDDKVAITSGNTSNVGVHVHASEIQSF			
	KGFMPGLIMPEDFTWNFRLQYTVNSRVKSDNITDI			
	QGGQGQLDLSTINYNLGHGTSNYSYGNSNAIDFEA			
	FFPGSVDSKTDKTQINGITPIGYGGSYSNSINVKTG			
	INQHQEKEVNSQAKIYQEHGKEGNVKGISHTIQNN			
	ANAAGIETVGIGELIVKKQDKFIKPAIANPFKLSKDG			
	VSGNVGSKISDTLDEIGQIAANIRALPKLGISGLIDA			
	KITKAALFDKKEPYEMTFKNIDQDGYTTIENLAIAKIR			
	TEDGSAQVVGGSPVRTTVFEIIVQDGNDNITDVKAELIG			
	KLDGTEVRVNLVSRENQGKAKLYSHIKKKNPFETG			
	KEGGTNTVEGULVENTENFTSGETNVKKDGKDYND			
	GRRPEVVSDVNLLANGGIKTIKVQVISDNKWTKIFNP			
	QIEGKGKIETVVKKRREITVGAPLETVAGNEGIGYTV			
	KVKTAEGPVIIGEEIKVIKGGVVYILYDQNNKKGTQRT			
	NSSNNFWTIHGAGDIEAKGQVVQKVVEELIKVGVVT			
	HQDNNIKTQAGINSSQFIVGTFTSNKSKVEADNNENQ			
	MGIKFIVLQGANEGENVTEIDIKNSTKTFENIKPFDIEG			
	KGEKIEVKNINDYDVNIKIEGNTKNIIETIKTTAKQENI			
	KEDKDNDKVKGNNEKTGIRPEIVTGTTNNINQILVLN			
	ESNNTTHTWAGLDKNGGVVEIELIRVKGGTHTHNVD			
	GNIGILIVTNKIVTEPTVEIKPSRPKDDFNTDVPNSKV			
	VRPPPPLPKTNSNNKADDDSKDNKTRKNPPLREKLPIT			
	GMRTIISWTIFIGILGYILIRLRRFNS			

**Table-2:** Antigenicity, allergenic, and solubility characteristics of selected *S. aureus* proteins.

*S. aureus* protein PDB ID	Name of the protein	Number of amino acids	Stability index	Predicted scaled solubility	Total number of negative/positive amino acid	Antigenicity	Pairwise multiple sequence alignment	

							Score	Possible cross-reaction with the host
1amx	Collagen Binding domain of adhesin	1183	16.89 (stable)	0.579 – Soluble, pI: 7.380	(Asp+Glu): 180/(Arg+Lys): 166	The sequence is probable allergen	−141	Non-host interacting
1yn3	Extracellular adherence protein	476	20.27 (stable)	0.905 Highly soluble, pI: 10.010	(Asp+Glu): 35/(Arg+Lys): 81	The sequence is probable allergen	10	Host interacting
1nu7	Staphylocoagulase thrombin complex Chain D	660	25.66 (stable)	0.487 – Soluble, pI: 9.660	(Asp+Glu): 89/(Arg+Lys): 93	The sequence is probable allergen	−154	Non-host interacting
2b71	Teichoic acid	327	21.68 (stable)	0.527 – Soluble, pI: 9.750	(Asp+Glu): 38/(Arg+Lys): 54	The sequence is probable allergen	−57	Non-host interacting
1edk	Protein A domain B	508	51.46 (unstable)	0.894 – Soluble, pI: 4.530	(Asp+Glu): 79/(Arg+Lys): 71	The sequence is probable non-allergen	63	Host interacting
2gi9	Protein GB1	593	20.50 (stable)	0.899 – Soluble, pI: 4.390	(Asp+Glu): 100/(Arg+Lys): 74	The sequence is probable allergen	101	Host interacting
1n67	Clumping factor A precursor	933	49.93 (unstable)	0.702 – Soluble, pI: 4.850	(Asp+Glu): 211/(Arg+Lys): 48	The sequence is probable non-allergen	−154	Non-host interacting
2noj	Extracellular fibrinogen binding complex	109	13.31 (stable)	0.758 – Soluble, pI: 10.780	(Asp+Glu): 6/(Arg+Lys): 23	The sequence is probable non-allergen	−206	Non-host interacting
1d20	Collagen binding protein Chain B	1183	16.89 (stable)	0.653 – Soluble, pI: 6.480	(Asp+Glu): 180/(Arg+Lys): 166	The sequence is probable allergen	−367	Non-host interacting

Of all the MHC I-binding peptides initially screened from *S. aureus*, only nine strong binders, collagen binding protein chain B (ID 1d20), extracellular fibrinogen binding complex (ID 2noj), clumping factor A precursor (ID 1n67), staphylocoagulase thrombin complex chain D (ID 1nu7), collagen binding domain of adhesin (ID 1amx), teichoic acid (ID 2b71), protein A domain B (ID 1edk), extracellular adherence protein (ID 1yn3), and protein GB1 (ID 2gi9) were chosen for further detailed analysis. These proteins underwent multiple sequence alignments to assess their similarity to MHC Class I molecules, and the selection was based on their alignment scores. The selected *S. aureus* proteins along with their alignment scores are listed in Table-2. The chosen peptides were further evaluated for their resemblance to self-antigens, as determined by their alignment and antigenicity scores, to check their ability to induce a cytotoxic T-cell response while avoiding the onset of autoimmunity. Pairwise sequence alignment was done to identify epitopes within the *S. aureus* peptides that the host immune system might recognize as self, while stability and solubility checking is important for the practical use of peptides in vaccines.

Any peptide with a scaled solubility value (QuerySol) above the population average (PopAvrSol) of 0.45 was considered highly soluble compared with a reference protein. This benchmark was instrumental in distinguishing peptides with favorable solubility characteristics for vaccine development, and any protein with a lower solubility value is predicted to be less soluble [37]. The solubility values are given in Table-2.

Peptides 1d20, 2noj, 1n67, 1nu7, 1amx, and 2b71 (listed in order of increasing sequence alignment scores) were recognized as promising candidates for eliciting a robust host immune response when bound to the MHC I, potentially avoiding autoimmune reactions. In addition, our allergenicity predictions using AllerTOP suggested six out of the nine selected peptides as potential allergens, excluding 1edk, 1n67, and 2noj. By integrating sequence alignment and allergenicity predictions, we identified peptides 1d20, 2noj, 1n67, 1nu7, 1amx, 2b71, and 1edk as likely to elicit a cytotoxic T-cell response. All selected peptides were predicted to be stable, except 1edk, and soluble, as detailed in Table-2. Among the peptides studied, 1n67 is distinguished by its high negative charge relative to the others. It demonstrates a favorable solubility score of 0.702 and a low molecular weight of 97,058 Daltons. The peptide sequence includes a domain spanning 933 amino acids, containing 211 negatively charged residues (Asp + Glu) and 48 positively charged residues (Arg + Lys). With an instability index of 49.93, this peptide is classified as unstable. It has an aliphatic index of 45.88 and a grand average of hydropathicity of −1.083.

### Molecular docking simulations to determine the binding capacity of S. *aureus* peptides to MHC I, CD8+, and TCR

**Figure-1:**
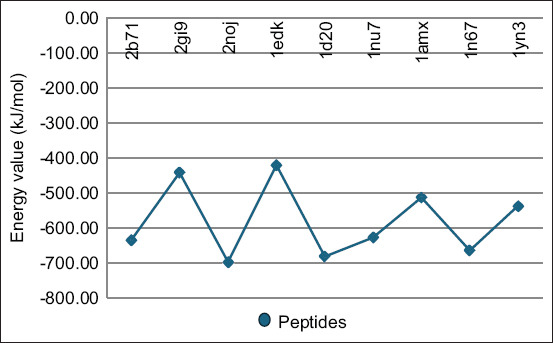
The energy value for docking analysis of the selected *Staphylococcus aureus* proteins with major histocompatibility complex class I.

**Figure-2:**
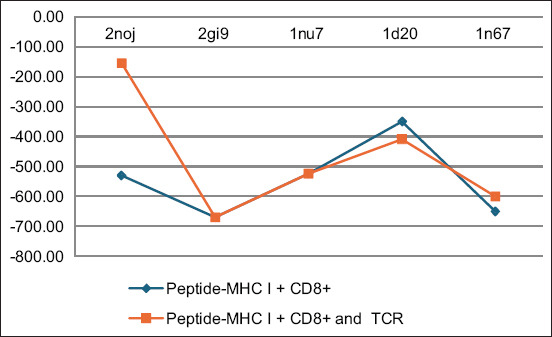
The energy value for docking analysis of *Staphylococcus aureus* proteins – major histocompatibility complex class I complex with CD8+ and T-cell receptor.

The molecular docking simulations provided insights into the interactions, binding energies, and potential stability of complexes formed between MHC I, CD8+, TCR, and selected *S. aureus* peptide epitopes. The energy values derived from these simulations are depicted in Figures-1 and 2. Figure-1 illustrates the energy values for the docking of selected *S. aureus* peptides with MHC I, whereas Figure-2 displays the energies for complexes involving *S. aureus* peptides MHC I with CD8+ and peptides - MHC I with CD8+ and TCR, highlighting the potential for stable complex formation.

Analysis of the *S. aureus* peptides docked with the active site of MHC I revealed that peptides with IDs 2noj, 1d20, 1n67, 2b71, 1nu7, 1yn3, 1amx, 2gi9, and 1edk demonstrated binding energy values of −698.1, −683, −665, −635, −628.1, −538, −514.1, −443, and −421.0, respectively, indicating their potential to be strong binders.

**Figure-3:**
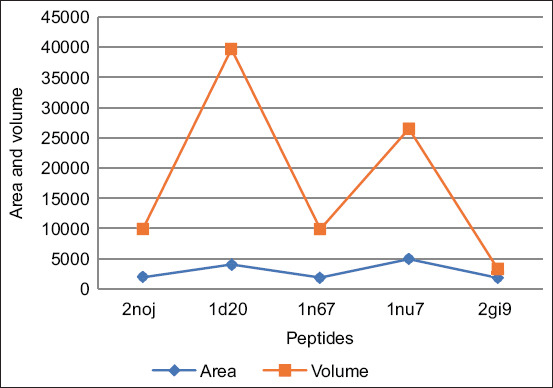
The area and volume of binding pockets as analyzed by computer atlas of surface topography of proteins.

The peptides bound with the lowest energy were considered the best among the studied peptides. The peptide ID 2gi9 – MHC I complex, when docked with CD8+ and the TCR, exhibited the lowest energy value of −669.9 KJ/mol, suggesting the formation of a highly stable complex, followed by 1n67, 1nu7, 1d20, and 2noj with energy values of −600, −524.1, −409, and −155.1, respectively. However, the peptide complexes of 1yn3, 1amx, and 2b71 - MHC I displayed lower ability to bind to the CD8+ and TCR complex. Further analysis using CASTp explored the binding pockets’ dimensions, both in terms of area and volume, across the various docked complexes. The analysis revealed that the area ranged between 2500 and 4000 and volume between 9000 and 400000. The areas and volumes of these binding pockets, as shown in Figure-3, ranged significantly, indicating diverse binding capacities among the peptides. Notably, peptide 1d20 exhibited the largest area (measuring 4043) and volume (39688) of binding pockets, suggesting its strong potential to elicit immunological responses due to the presence of significant binding pockets.

From the comprehensive analyses conducted through various simulations, the peptide IDs 2noj, 1d20, 2gi9, and 1n67 emerged as the most promising candidates for further vaccine development and as potential elicitors of T-cell-mediated immunity.

## Discussion

The pursuit of a vaccine against *S. aureus* stands as a crucial frontier in the battle against bacterial infections, especially in the light of resistance to current antibiotic therapies [38]. The present study significantly identifies potential *S. aureus* peptides and their epitopes for stimulating CD8+ T-cell mediated immunity through MHC I molecules. The intricate pathogenicity of *S. aureus*, along with its ability to evade the host immune response, poses a considerable challenge in vaccine development [13, 38]. This research underscores the complexity of identifying immunogenic peptides by employing the SVMTriP tool for epitope prediction and integrating alignment scores, antigenicity, stability, solubility, and molecular docking simulations. Although the specific mechanisms through which *S. aureus* peptides might offer protection have not yet been fully elucidated, emerging evidence supports the notion that antigens capable of activating CD8+ T-cells could pave a promising pathway toward an effective defense against *S. aureus* infections [13, 15, 38]. Identification of antigens that are able to stimulate a CD8+ T response through MHC Class I molecules is pivotal in creating an effective vaccine for *S. aureus*, particularly for infections where the bacteria persist intracellularly [13, 15, 39, 40].

*In silico* screening serves as a crucial preliminary step before experimental validation, streamlining the identification of viable antigenic peptides capable of eliciting CD8+ T-cell responses. This approach significantly reduces the time and resources required for conducting wet laboratory experiments [41, 42]. This approach is also particularly valuable in the context of infectious diseases, where rapid vaccine development is crucial [43].

Nine potential secretory and surface peptides from *S. aureus* were identified through the initial screening. High-affinity binding of these peptides to MHC I molecules is crucial for effective antigen presentation and CD8+ T-cell activation. This step is crucial for mounting an immune response to intracellular *S. aureus*. To prevent autoimmunity, it is crucial to focus on peptides that differ significantly from self-antigens. By minimizing adverse effects, this method assesses the antigen’s potential to induce an immune response [13, 38].

Subsequently, the nine selected peptides were subjected to further analysis, focusing on antigenicity, stability, solubility, and molecular docking simulations. From this comprehensive evaluation, four peptides namely, 2noj, 2gi9, 1d20, and 1n67 emerged as leading candidates because of their capacity to induce robust CD8+ T-cell responses on presentation by MHC I. The selection of these four peptides was based on several key criteria, such as low allergenic potential, which ensures that the peptides are unlikely to cause allergic reactions in recipients; non-self-characterization, which ensures that the peptides do not closely mimic self-antigens to avoid triggering autoimmunity; solubility and stability, which are crucial for the peptides to remain effective under physiological conditions, facilitating their use in vaccine formulations and storage; and high immunogenicity, which ensures that the peptides possess a strong ability to elicit an immune response [44, 45]. Solubility influences not only the manufacturability of a vaccine but also its immunogenicity, as highly soluble peptides are more likely to maintain their structural integrity and interact effectively with the immune system [45]. Adding solubility criteria to the vaccine candidate selection process increases its stability. The distinction between virulence and non-virulence factors is crucial in vaccine design. By employing this approach, the risk of adverse reactions is reduced and a safer vaccination process is facilitated [44, 45].

Although protein A and membrane proteins contribute significantly to the virulence of *S. aureus*, the novel immune elicitors discovered in this study focus on epitopes that diverge from major virulence factors. This strategy potentially minimizes adverse reactions and offers a safer pathway for vaccine development [44]. Molecular docking simulations revealed the likelihood of stable complex formation between selected peptides, MHC I molecules, CD8+ co-receptors, and TCR. The simulations suggest the immunogenicity potential of peptide-MHC I complexes, as indicated by their binding energy values. Identification of peptides with strong binding to MHC I and favorable interactions with CD8+ and TCR suggests a robust mechanism for T-cell activation and response [32, 33].

The immune response is marked by intricate complexity due to its diverse nature. The varying sizes of peptide binding pockets indicate a broad scope for their immune receptor interactions. This diversity underscores the complexity of the immune response [35]. 2noj, 2gi9, 1d20, and 1n67, with dimensions and areas suitable for binding pockets, were identified as potent immune elicitors.

Thorough characterization of the vaccine component’s immunogenicity, molecular weight, charge distribution, physioco-chemical properties and stability is essential. Such in-depth characterization is crucial for assessing the immunogenic potential and stability of vaccine components [36, 46, 47]. The peptide 1n67 has more negatively charged amino acids than positively charged ones, which typically hinder cell membrane interactions and impede internalization into cells. This peptide, identified as a virulence factor [46–48], merits further investigation due to its unique charge characteristics [47–49]. CD8+ cells can be activated by peptide 1n67 through MHC Class I, making it a suitable candidate for a vaccine. This hypothesis relies on the peptide’s physicochemical characteristics and the function of charge in facilitating interactions between the peptide and the cell membrane. The understanding of immunological responses to pathogens is evolving toward designing vaccines that activate CD8+ cells through MHC I, with MHC I’s capability to present numerous antigenic peptides being crucial for initiating cellular immune responses. This insight is critical for developing subunit and synthetic peptide vaccines targeting the cellular arm of the immune system [50–53].

The use of SVMTrip and other *in silico* tools in this study highlights the potential of computational biology to streamline the initial stages of vaccine development. By identifying antigenic epitopes capable of engaging the immune system, these methods contribute to the broader goal of creating effective immunotherapies and diagnostics for infectious diseases [52].

Membrane proteins are particularly prioritized as vaccine candidates because of their ability to initiate a cascade of immune responses [27]. Our study confirmed that peptide 2noj, a surface protein, is exceptionally strong in binding to MHC Class I molecules. In the docking simulation, specific peptides were shown to bind with MHC Class I molecules. We selected peptide-MHC complexes capable of inducing CD8+ T-cell responses. The concentration of antigenic peptides and the affinity between TCR and the antigen-MHC complex play crucial roles in T cell activation. CD8+ naive T-cells are triggered to become effector cytotoxic T lymphocytes on being stimulated by inflammatory signals and antigen-presenting cells [50, 53].

ASL treatment shows promise in combating *S. aureus* infections due to its ability to induce cell-mediated immunity [17]. Most research on the therapeutic effectiveness of ASL has focused on animal models, especially bovine mastitis. The mixture of *S. aureus* antigens in ASL probably stimulates a T-cell response through MHC Class I molecules [16-18]. The identified peptides, specifically IDs 2noj, 1d20, 2gi9, and 1n67, which are likely to be found in ASL are proposed to induce a CD8+ T-cell response through MHC Class I.

## Conclusion

By examining *S. aureus* peptide antigens and their relationship with MHC Class I molecules and CD8+ T-cells, our study proposes a comprehensive method for discovering *S. aureus* peptide epitopes that elicit CD8+ T-cell-mediated immunity. *In silico* tools such as epitope prediction, solubility assessment, and molecular docking simulations have led to a more profound comprehension of immunological interactions. The analysis has revealed several promising candidates, such as peptides IDs 2noj, 1d20, 2gi9, and 1n67, providing hope for creating an effective vaccine against *S. aureus* and shedding light on the immune response triggered by ASL. These findings provide a solid basis for further experimentation and potential development of an immune response elicitor against a powerful pathogen. While *in silico* findings provide valuable insights, practical vaccine formulations require rigorous *in vitro* and *in vivo* evaluations. To confirm immunogenicity, efficacy, and safety of identified peptides against *S. aureus*, detailed evaluations are necessary. The immunogenicity, efficacy, and safety of the identified peptides must be validated through comprehensive testing.

## Authors’ Contributions

JRTP: Designed the study and wrote and revised the manuscript. GVB: Designed the study, wrote and revised the manuscript, and analyzed data. KB: Scrutinized the data and edited and reviewed the manuscript. JRTP, GVB, KB, and PS: Extracted data and reviewed the manuscript. All authors have read, reviewed, and approved the final manuscript.
